# Where should stepped-wedge designs be placed in the evidence hierarchy? Using the “within-wedge” analysis approach to generate evidence of possible bias

**DOI:** 10.1186/1472-6963-14-S2-P54

**Published:** 2014-07-07

**Authors:** Terry Haines, Karla Hemming, Alan Girling, Anne-Marie Hill, Max Bulsara, Jon Deeks

**Affiliations:** 1 Physiotherapy Department, Monash University Frankston, Victoria, Australia; 2 Allied Health Research Unit, Monash Health, Clayton, Victoria, Australia; 3 School of Health and Population Sciences, University of Birmingham, Birmingham, UK; 4 Physiotherapy Department, University of Notre Dame, Fremantle, Western Australia, Australia; 5 Institute for Health Research, University of Notre Dame, Fremantle, Western Australia, Australia

## 

The stepped wedge research design is becoming increasingly popular, particularly in the field of implementation science. It is a form of cluster randomised controlled trial with unidirectional cross-over (normally from control to intervention). This trial design may be biased however because the effect of calendar time is unbalanced between control and intervention periods. Hence there is concern that this design may produce biased results compared to using a parallel cluster randomised controlled trial. Authors have previously compared these two designs on the grounds of data collection burden and cost. However, it is arguably more important to compare these designs in terms of whether they are equally likely to generate results that are free from bias. This paper discusses the potential sources of bias relevant to these designs, examines how empirical evidence of bias has previously been generated, and then outlines the “within-wedge” analysis approach - a new method for generating evidence of potential bias in the stepped-wedge design.

There have been four strategies previously used to generate empirical evidence of bias with different research designs. These include; i) direct comparison of results from trials that have used different designs to answer the same question, ii) meta-epidemiology, iii) resampling from existing studies, and iv) resampling from custom-developed datasets. Each approach has strengths and limitations in the evidence they can generate. For example, approach i) requires minimal variation in the study designs and populations in order to minimise confounding when making comparisons, while approach ii) requires data from large numbers of studies to be gathered.

The within-wedge analysis approach is a variation on approach i) made possible through the realisation that data from a parallel cluster randomised trial is hidden within a stepped wedge design. The two effect estimates generated (one from the stepped-wedge design, one from the parallel cluster trial design) can be compared in a ratio of ratios. The within-wedge analysis approach has an advantage over approach i) in that many study characteristics are held constant, and an advantage over approach ii) in that this approach can be applied to individual studies (and to multiple outcomes within individual studies). Meta-regression can be applied to within-wedge analysis outcomes to identify situations that may make stepped-wedge designs more or less prone to bias. We recommend that the within-wedge analysis reported as a secondary analysis from stepped-wedge designs in future.

